# Prevalence and factors associated with utilisation of postnatal care in Sierra Leone: a 2019 national survey

**DOI:** 10.1186/s12889-022-12494-5

**Published:** 2022-01-14

**Authors:** Quraish Sserwanja, Lilian Nuwabaine, Kassim Kamara, Milton W. Musaba

**Affiliations:** 1 Programmes Department, GOAL Global, Arkaweet Block 65 House No. 227, Khartoum, Sudan; 2 School of Nursing and Midwifery, Aga Khan University, Kampala, Uganda; 3 National Disease Surveillance Programme, Ministry of Health and Sanitation, Free town, Sierra Leone; 4 Department of Obstetrics and Gynaecology, Mbale Regional Referral and Teaching Hospital, Mbale, Uganda; 5 Department of Obstetrics and Gynaecology, Busitema University, Tororo, Uganda

**Keywords:** Postnatal care, Sierra Leone, Women, DHS, Utilisation

## Abstract

**Background:**

Within Sub-Saharan Africa, some countries still report unacceptably high rates of maternal and perinatal morbidity and mortality, despite improvements in the utilisation of maternity care services. Postnatal care (PNC) is one of the recommended packages in the continuum of maternity care aimed at reducing maternal and neonatal mortality. This study aimed to determine the prevalence and factors associated with PNC utilisation in Sierra Leone.

**Methods:**

We used Sierra Leone Demographic and Health Survey (UDHS) 2019 data of 7326 women aged 15 to 49 years. We conducted multivariable logistic regression to determine the factors associated with PNC utilisation, using SPSS version 25.

**Results:**

Out of 7326 women, 6625 (90.4, 95% CI: 89.9–91.2) had at least one PNC contact for their newborn, 6646 (90.7, 95% CI: 90.2–91.5) had a postnatal check after childbirth and 6274 (85.6, 95% CI: 85.0–86.6) had PNC for both their babies and themselves. Delivery by caesarean section (aOR 8.01, 95% CI: 3.37–19.07), having a visit by a health field worker (aOR 1.80, 95% CI: 1.46–2.20), having had eight or more ANC contacts (aOR 1.37, 95% CI: 1.08–1.73), having tertiary education (aOR 2.71, 95% CI: 1.32–5.56) and having no big problems seeking permission to access healthcare (aOR 1.51, 95% CI: 1.19–1.90) were associated with higher odds of PNC utilisation. On the other hand, being resident in the Northern (aOR 0.48, 95% CI: 0.29–0.78) and Northwestern regions (aOR 0.54, 95% CI: 0.36–0.80), belonging to a female headed household (aOR 0.69, 95% CI: 0.56–0.85) and being a working woman (aOR 0.66, 95% CI: 0.52–0.84) were associated with lower odds of utilizing PNC.

**Conclusion:**

Factors associated with utilisation of PNC services operate at individual, household, community and health system/policy levels. Some of them can be ameliorated by targeted government interventions to improve utilisation of PNC services.

**Supplementary Information:**

The online version contains supplementary material available at 10.1186/s12889-022-12494-5.

## Introduction

Annually, sub-Saharan Africa records the highest maternal deaths at 500 per 100,000 live births, and newborn deaths at 34 per 1000 live births [1], which accounts for 38% of the global neonatal deaths. Within the same region, Sierra Leone is still among the top three countries with the highest maternal mortality ratio (MMR) [2, 3]. The 2019 Sierra Leone Demographic and Health Survey (SLDHS) showed that the maternal mortality ratio (MMR) was 717 deaths per 100,000 live births, meaning for every 1000 live births, about seven women (7.17) died during pregnancy, childbirth or within 6 weeks of childbirth [4]. This MMR is over 10 times higher than the SDG 3 target of 70 deaths per 100,000 live births by 2030 [5]. SLDHS further shows that neonatal mortality ratio (NMR) stands at 31 per 1000 live births, down from 39 per 1000 live births in 2013 [4].

Timely access to maternal health services is an effective intervention aimed at reducing pregnancy and childbirth related deaths [1, 6]. Over the decade, progress has been reported in ensuring increase in the continuum of maternal healthcare utilisation [7]. However, the progress has been mainly with skilled antenatal care attendance and facility-based deliveries with little progress regarding PNC utilisation [7, 8]. Yet,the immediate postpartum period is critical for the survival of both mothers and newborns because that is when most physiological adaptations occur [1, 5]. In this regard, receipt of timely postnatal care (PNC) is widely used to track progress towards national and international maternal child health goals [9, 10]. PNC is one of the recommended packages in the continuum of maternity care, aimed at reducing maternal and neonatal mortality [1, 11]. This service consist of care given to mothers and neonates right after delivery and up to 6 weeks of postpartum with the aim of ensuring optimum health for the mother and the infant [1]. Timely PNC enables health workers to detect, follow, and quickly manage complications of both the mother and newborn [12]. In addition, timely PNC accords an opportunity to receive health information and support for positive practices such as exclusive breastfeeding, maternal and newborn danger signs, care of the newborn that are key to maternal and child health and survival [13]. Therefore, WHO recommends the first postnatal check to occur within the first 24 h of childbirth and then, at least three other postnatal visits are arranged for all mothers and newborns, on day 3, between the 1st and 2nd weeks, and 6 weeks after childbirth [1, 7]. However, about 63% of mothers and 48% of newborns globally utilise PNC within the recommended timeframe [1] and less than 25% of newborns in less developed countries receive PNC within 2 days of delivery [1].

Although, the benefits of PNC in reducing maternal and neonatal deaths have been well documented, postnatal services have the lowest median national coverage of all the interventions on the continuum of maternal and child healthcare [14, 15]. Sierra Leone’s long civil war and Ebola epidemic left the health system fragile and overwhelmed with inadequate skilled health personnel having low and irregular remuneration [16], increasing demand and stock-outs of crucial medical supplies and equipment leading to patients having to pay for services that are meant to be free [17, 18]. Secondary and tertiary care in Sierra Leone is provided by 14 district and regional governmental hospitals and four tertiary referral hospitals which are all located in the Western Area Urban District [19]. The country’s nurse density is one of the world’s lowest having approximately 0.2 nurses and midwives per 1000 people [16].

Despite several government interventions to improve maternal and child health services such as Free Health Care Initiative (FHCI) introduced in 2010 to address the issue of cost as a barrier to utilisation of maternal healthcare services [20], not much has been achieved because the MCH indicators have remained unacceptably high. In order to reduce the high maternal and perinatal mortality, there is need for up to date and evidence based information to guide the formulation and implementation of effective strategies. However, there is dearth of information about the level and determinants of PNC utilisation for both mother and baby pair. Prior studies on the same subject by Koroma et al., and Sharkey et al. did not use a nationally representative sample, and they focused on antenatal care (ANC), skilled birth attendance and health facility delivery utilization [21, 22]. On the other hand, Jalloh et al. analysed data from 2008 to 2013 Sierra Leone Demographic Health Surveys (SLDHS) and evaluated wealth related inequity in the utilisation of maternal health services [17]. The study showed increment in PNC utilisation and that PNC reviews were more equally distributed than ANC visits. However, the study was only able to model factors associated with institutional delivery and so did not provide factors associated with PNC utilisation [17]. Therefore, the current study aimed to determine the prevalence and factors associated with PNC utilisation in Sierra Leone. This is among the first studies to use data from the most recent nationally representative survey to determine the level and determinants of PNC utilisation by both the mother and newborn pair in Sierra Leone.

## Methods

### Data source

This study used secondary data from the 2019 Sierra Leone Demographic and Health Survey (SLDHS). Data were accessed from MEASURE DHS database at http://dhsprogram.com/data/available-datasets.cfm. SLDHS was a nationally representative cross-sectional survey implemented by Statistics Sierra Leone (Stats SL) with technical assistance from ICF intern through the DHS Program and funded by the United States Agency for International Development (USAID). The Demographic and Health Survey datasets are freely available to the public though researchers must register with MEASURE DHS and submit a request before accessing them.

### Study sampling and participants

The 2019 SLDHS samples were selected using a stratified, two-stage cluster sampling design that resulted in the random selection of 13,872 households [4]. Detailed sampling procedures were published in the final report [4]. DHS uses different questionnaires; household questionnaire collects data on household environment, assets and basic demographic information of household members while women’s questionnaire collects data about women’s reproductive health, domestic violence and nutrition indicators. The individual record (IR) file used in this study contains all the collected data in the women’s questionnaire for de facto women plus some variables from the household questionnaire. This secondary analysis included women aged 15 to 49 years who had a live birth within 5 years preceding the survey and were either permanent residents or slept in the selected household the night preceding the survey. Out of the total weighted sample of 15,574 women in the data set, only 7326 had given birth within 5 years preceding the survey (Table 1). Of the 7326 women, 126 women had missing data leading to a total of 7200 women for logistic regression analysis (Table 3).

**Table 1:** Socio-demographic characteristics of women in Sierra Leone as per the 2019 SLDHS

Characteristics	*N* = 7326	%
**Age**		
15 to 19	598	8.2
20 to 34	4830	65.9
35 to 49	1898	25.9
**Residence**		
Urban	2795	38.1
Rural	4531	61.9
**Region**		
Western	1479	20.2
Eastern	1542	21.0
Northwestern	1380	18.8
Northern	1433	19.6
Southern	1492	20.4
**Religion**		
Islam	5766	78.7
Christianity and others	1560	21.3
**Sex household head**		
Male	5520	75.3
Female	1806	24.7
**Household Size**		
7 and above	3319	45.3
Less than 7	4007	54.7
**Working status**		
Not working	1683	23.0
Working	5643	77.0
**Marital status**		
Not married	1329	18.1
Married	5997	81.9
**Education Level**		
No Education	3857	52.7
Primary Education	1033	14.1
Secondary Education	2214	30.2
Tertiary	221	3.0
**Wealth Index**		
Poorest	1587	21.7
Poorer	1551	21.1
Middle	1487	20.3
Richer	1441	19.7
Richest	1259	17.2
**Parity**		
1	1989	27.1
2–4	4015	54.8
5 and above	1323	18.1
**Exposure to newspapers**		
No	6921	94.5
Yes	405	5.5
**Exposure to Radio**		
No	4224	57.7
Yes	3102	42.3
**Exposure to TV**		
No	5579	76.2
Yes	1747	23.8
**Internet use**		
No	6586	89.9
Yes	740	10.1
**Permission to access healthcare**		
Big problem	1827	24.9
Not big problem	5499	75.1
**Distance to health facility**		
Big problem	3454	47.1
Not big problem	3872	52.9
**ANC timing** ^**a**^		
Above first trimester	4000	55.4
Within first trimester	3214	44.6
**ANC attendance**		
8 contacts and above	1610	22.0
Less than 8 contacts	5715	78.0
**Method of birth** ^**b**^		
Caesarean section	324	4.4
Vaginal	6988	95.6
**Skilled birth attendance**		
Yes	6468	88.3
No	858	11.7

^a^missing 113 (1.5%) respondents

^b^missing 13 (0.2%) respondents

### Variables

#### Dependent variables

The outcome variable was PNC utilisation which was considered as atleast one postnatal check for both the mother and the neonate within the postpartum period and was constructed into a binary variable coded as one (1) if the mother and neonate utilised PNC and zero (0) if no PNC utilisation for both mother and the neonate.

#### Independent variables

This study included determinants of ANC initiation timing and frequency based on evidence from available literature and data [1, 7, 11, 14]. Twenty-one explanatory variables were used in this study. Maternal age was categorised as; (15–19 years, 20–34 years and 35–49 years). Wealth index is a measure of relative household economic status and was calculated by UDHS from information on household asset ownership using Principal Component Analysis, which was further categorised into poorest, poorer, middle, richer and richest quintiles [23]. Place of Residence was categorised into urban and rural.

Region was categorised into four; Northern, Eastern, Southern, Western and Northwestern while level of Education was categorised into no education, primary education, secondary and tertiary education. Household Size was categorised as less than seven members and seven and above members (based on the dataset average of seven members per household). Sex of household head was categorised as male or female, working status categorized as: not working and working while marital status as married (this included those in formal and informal unions) and not married. Religion was categorised as Muslims and Christians and others, problems seeking permission and distance to health facility were categorised as big problem and no big problem while exposure to mass media and internet use (TV, radio, and newspapers) were categorized as yes and no. In the questionnaire, seeking permission to access healthcare and distance to health facility had three original responses: no problem, no big problem and big problem. However, none of the study participants reported no problem hence we only had two responses. Skilled birth attendance was categorised as yes and no, place of child birth as home and health facility and method of delivery as caesarean section and vaginal.

### Statistical analysis

Analysis was carried out based on the weighted count to account for the unequal probability sampling in different strata and to ensure representativeness of the survey results at the national and regional level. In order to account for the multi-stage cluster study design, complex sample package of SPSS (version 25.0) statistical software was used. We used SPSS version 25.0 statistical software complex samples package incorporating the following variables in the analysis plan to account for the multistage sample design inherent in the DHS dataset: individual sample weight, sample strata for sampling errors/design, and cluster number [24–26]. Use of complex samples package ensures that the sample design is incorporated into the analysis leading to accurate and reliable results.

Before multivariable logistic regression, each exposure/predictor (independent variable) was assessed separately for its association with PNC utilisation using bivariable logistic regression and we presented the crude odds ratio (COR), 95% confidence interval (CI) and *p*-values. Independent variables associated with PNC utilisation with a *p*-value ≤0.25 at the bivariable level, and not strongly collinear with other independent variables (considered variance inflation factor (VIF) less than 2.5) [27] with other independent variables were considered for multivariable logistic regression to assess the independent effect of each variable on the PNC utilisation. Residence, wealth index, skilled birth attendance and place of delivery were not included in the multivariable model because they had VIFs above 2.5 with many other independent variables. Adjusted odds ratios (AOR), 95% confidence intervals (CI) and *p*-values were calculated with statistical significance level set at *p*-value < 0.05. Sensitivity analysis was done including the variables that had VIF above 2.5 but less than 5 and results are shown in Supplementary file 1.

## Results

A total of 7326 women were included in the analysis (Table 1). Out of 7326 women, 6625 (90.4, 95% CI: 89.9–91.2) had their babies have at least a PNC contact, 6646 (90.7, 95% CI: 90.2–91.5) had a postnatal check after childbirth and 6274 (85.6, 95% CI: 85.0–86.6) had PNC for both their babies and themselves as shown in Table 2. Majority of the women had less than eight ANC contacts (78.0%), had skilled birth attendance (88.3%) were residing in rural areas (61.9%), were Muslims (78.7%), had no education (52.7%), resided in male headed households (75.3%), were married (81.9%), working (77%) and aged between 20 and 34 years (65.9%). Mass media exposure was limited with 57.7% of women not exposed to radio, 76.2% not exposed to TV, 89.9% not using internet and 94.5% not exposed to newspapers. The mean age and household size were 28.97 + 7.25 and 6.93 + 3.45 respectively. Regarding content of PNC, 4895 women (66.8, 95% CI: 65.7–67.8) had all the contents of PNC check that included: examining the code, temperature being taken, breastfeeding and newborn danger signs counseling and having a practical breastfeeding session with the health provider as shown in Supplementary file 2.

**Table 2:** Utilisation of postnatal care

Service	Frequency *N* = 7326	%	95% CI
Maternal PNC at health facility discharge^a^	5706	92.3	91.7–93.0
Maternal PNC after discharge from health facility	2715	37.1	35.9–38.1
Maternal PNC (any of the above)	6646	90.7	90.2–91.5
Neonatal PNC at health facility discharge^a^	5735	92.8	92.1–93.4
Neonatal PNC after discharge from health facility	3329	45.5	44.6–46.8
Neonatal PNC (any of the above)	6625	90.4	89.9–91.2
Both maternal and neonatal PNC	6274	85.6	85.0–86.6

^a^Misssing 1143

### Factors associated with PNC utilisation

After adjusting for other variables, factors that were positively associated with PNC utilisation as shown in Table 3 were; delivery by caesarean section (aOR 8.01, 95% CI: 3.37–19.07), having a visit by a health field worker (aOR 1.80, 95% CI: 1.46–2.20), having had eight or more ANC contacts (aOR 1.37, 95% CI: 1.08–1.73), having tertiary education (aOR 2.71, 95% CI: 1.32–5.56) and having no big problems seeking permission to access healthcare (aOR 1.51, 95% CI: 1.19–1.90). Exposure to radio (aOR 1.24, 95% CI: 0.99–1.54) was marginally associated with more odds of PNC utilisation compared to non-exposure. On the other hand, being resident in the Northern (aOR 0.48, 95% CI: 0.29–0.78) and Northwestern regions (aOR 0.54, 95% CI: 0.36–0.80), belonging to a female headed household (aOR 0.69, 95% CI: 0.56–0.85) and being a working woman (aOR 0.66, 95% CI: 0.52–0.84) were associated with lower odds of utilizing PNC compared to being a resident in the Western region and not being a working woman.

**Table 3:** Factors associated with PNC utilisation in Sierra Leone as per the 2019 SLDHS

Characteristics	Crude model cOR (95% CI)	*P*-value	Adjusted model aOR (95% CI)	*P*-value
**Caesarean section**				
No	1		1	
Yes	7.10 (3.20–15.76)^a^	< 0.001	8.01 (3.37–19.07)^a^	< 0.001
**Skilled birth attendance**				
Yes	1			
No	5.86 (4.60–7.47)^a^	< 0.001	–	
**Visited by fieldworker**				
No	1		1	
Yes	1.77 (1.44–2.18)^a^	< 0.001	1.80 (1.46–2.20)^a^	< 0.001
**ANC frequency**				
Less than 8 contacts	1		1	
8 contacts and above	1.42 (1.12–1.81)^a^	0.004	1.37 (1.08–1.73)^a^	0.010
**ANC initiation timing**				
First trimester	1		1	
After first trimester	1.11 (0.93–1.32)	0.255	1.10 (0.91–1.32)	0.339
**Age**				
35 to 49	1			
20 to 34	1.26 (1.08–1.48)^a^	0.003	1.06 (0.87–1.29)	0.544
15 to 19	1.30 (0.98–1.73)	0.068	0.98 (0.66–1.43)	0.898
**Residence**				
Rural	1			
Urban	1.41 (1.08–1.84)^a^	0.011	–	
Region				
**Western**	1		1	
Southern	1.10 (0.72–1.66)	0.668	1.31 (0.84–2.04)	0.242
Northwestern	0.48 (0.33–0.72)^a^	< 0.001	0.54 (0.36–0.80)^a^	0.003
Northern	0.48 (0.31–0.74)^a^	0.001	0.48 (0.29–0.78)^a^	0.003
Eastern	0.72 (0.48–1.08)	0.110	0.83 (0.54–1.28)	0.400
**Religion**				
Christianity and others	1		1	
Islam	0.82 (0.66–1.02)	0.075	1.04 (0.84–1.30)	0.711
**Sex household head**				
Male	1		1	
Female	0.82 (0.68–0.99)^a^	0.047	0.69 (0.56–0.85)^a^	< 0.001
**Household Size**				
7 and above	1		1	
Less than 7	1.12 (0.93–1.35)	0.231	1.04 (0.86–1.26)	0.672
**Working status**				
Not working	1		1	
Working	0.61 (0.49–0.75)^a^	< 0.001	0.66 (0.52–0.84)^a^	0.001
**Marital status**				
Not married	1		1	
Married	0.85 (0.69–1.03)	0.095	0.96 (0.76–1.21)	0.732
**Education Level**				
No Education	1		1	
Primary Education	1.12 (0.91–1.38)	0.268	0.97 (0.77–1.23)	0.249
Secondary Education	1.49 (1.22–1.82)^a^	< 0.001	1.16 (0.90–1.48)	0.248
Tertiary	3.98 (2.17–7.30)^a^	< 0.001	2.71 (1.32–5.56)^a^	0.006
**Wealth Index**				
Poorest	1		–	
Poorer	1.27 (1.03–1.57)^a^	0.026		
Middle	1.66 (1.26–2.18)^a^	< 0.001		
Richer	1.77 (1.28–2.46)^a^	0.001		
Richest	2.05 (1.39–3.02)^a^	< 0.001		
Parity				
5 and above	1		1	
2–4	1.17 (0.99–1.38)	0.07	0.95 (0.78–1.17)	0.644
1	1.42 (1.17–1.74)^a^	< 0.001	1.06 (0.81–1.40)	0.668
**Newspapers’ exposure**				
No	1		1	
Yes	1.47 (0.90–2.38)	0.121	0.95 (0.55–1.66)	0.861
**Exposure to Radio**				
No	1		1	
Yes	1.31 (1.06–1.62)^a^	0.012	1.24 (0.99–1.54)	0.052
**Exposure to TV**				
No	1		1	
Yes	1.18 (0.93–1.50)	0.178	0.77 (0.60–1.00)	0.052
**Internet use**				
No	1		1	
Yes	1.76 (1.17–2.66)^a^	0.007	0.94 (0.59–1.51)	0.801
**Permission to access** healthcare				
Big problem	1		1	
Not big problem	1.51 (1.21–1.90)^a^	< 0.001	1.51 (1.19–1.90)^a^	0.001
**Distance to health facility**				
Big problem	1		1	
Not big problem	1.25 (0.99–1.59)	0.065	1.02 (0.78–1.33)	0.869
**Delivery place**				
Home	1		–	
Health facility	7.26 (5.74–9.19)^a^	< 0.001	–	

^a^significant at < 0.05

## Discussion

We found that 85.6% of both the women and their neonates had at least one PNC contact, and this was associated with both individual and health system/policy factors. Although, slightly lower than the 90% level of PNC coverage recommended by WHO [28], it is higher than the reported prevalence of 48.4% for Malawi, 63% for Zambia and 78.4% for Indonesia [29–31]. This level of utilisation is commendable and should be further supported by government interventions, aimed at providing free maternal and child health services through the FHCI project. However, with only 66.8% of women being able to have had all the contents of PNC check, there is need to focus on content and quality of PNC.

The odds of PNC utilisation were eight times higher among women that delivered by caesarean compared to those that delivered vaginally. This is not surprising because women who have operative deliveries are at higher risk of suffering a wide range of postoperative complications. Therefore, frequent return to the health facilities for checkup would be a mitigation strategy to minimize the perceived risks and in return get the opportunity to utilise PNC [30, 32]. It is also true that generally, patients that have undergone surgery are in most cases given extra attention by health personnel regarding awareness on the complications which come after delivery thus take the scheduled postnatal visits more seriously [33, 34].

Several studies conducted in Uganda, Ethiopia, Malawi, Zambia and Tanzania, have shown that increased frequency of ANC contacts is associated with higher odds of PNC utilisation [30, 31, 35–37]. Similarly, in this study the odds of PNC utilisation were higher among woman who had eight or more ANC contacts compared to their counterparts who had fewer. This may be attributed to the fact that regular contact with health workers during ANC, accords more opportunities for education and counselling about the need to seek for health care services during and after pregnancy [28, 31, 38]. Mothers who had at least one visit by a health field worker had higher odds of PNC utilisation, a finding similar to that of other studies done in similar contexts [39, 40]. The repeated contact with health workers during pregnancy through ANC services and visits by health field workers promote confidence and familiarity with the health system leading to increased trust in the health system [36]. This emphasizes the need to build capacity among field health workers, so that they are empowered to counsel women to seek PNC services at the community level in addition to strengthening the services in the health facilities.

Women in the Northern region had lower odds of PNC utilisation compared to those in the Western region. In Sierra Leone, the Western region has the largest concentration of health workers, is the most developed region, which also houses the capital and economic city of the country and hence has higher quality social amenities compared to other regions [41, 42]. Therefore, women in the Western region have easier access to health care facilities for PNC and are more likely to afford the direct and indirect costs involved in seeking PNC compared to those from the Northern region which is one of the poorest regions with inadequate skilled staff and infrastructure [21, 43, 44]. However, more studies are needed to explore these regional differences in the utilisation of PNC. The role of regional disparities in explaining PNC utilisation has also been documented in previous studies in Malawi, Tanzania and Ghana [31, 39, 45]. Women who had no big problems seeking permission to access healthcare had higher odds of utilizing PNC services. It is widely reported that empowering women to individually take decisions concerning their maternal health demands has greatly shown positive impact in the utilisation of services like PNC [37]. The influence of spouses and family members in the women’s decision making towards seeking health care services has been documented elsewhere as a key factor limiting utilisation of services like PNC [31, 46].

Women who were of working class, and those from female headed households had lower odds of utilising PNC services compared to their counterparts who were not working and those belonging to male headed households. This is not new because maternal employment has been shown by El-gilany et al. to negatively affect utilisation of maternal healthcare which is majorly attributed to unfavorable working conditions such as short or no maternal leave periods and long working hours which give mothers less or no time to seek these services [47, 48]. Male involvement in maternal healthcare has been shown to increase utilisation and positive outcomes due to better decision making, social and financial support [49, 50]. Hence, the lower odds of PNC utilisation among female headed households could be partly attributed to less social and financial strength these women have which affects their ability to seek PNC. Relatedly, women who had attained tertiary level education had higher odds of utilizing PNC services. This is no surprising because educated women are more likely to have safe and better employment opportunities, be more open to receiving new health related information positively hence more awareness about available health resources and more knowledge about health behaviors and the healthcare system which leads to better maternal literacy and informed healthcare decision-making abilities,access and utilization [6, 51, 52].

In the sensitivity analysis (Supplementary file 1), belonging to richer wealth quintile was significantly associated with increased utilisation of PNC services, a finding similar to studies done in Guinea, Zambia and Ethiopia [29, 34, 46, 53]. Wealth index being a proxy of financial status means that women in higher wealth indices can easily afford the direct and indirect costs involved in accessing quality and timely healthcare [54, 55]. Furthermore, women from wealthier households tend to be more enlightened and empowered hence have more decision making powers which enables them to have timely and more frequent healthcare access [54]. Given that Sierra Leone has free maternal healthcare services [42], our results suggest that, apart from the cost of health services, other economic factors play a key role in influencing PNC utilisation. However, studies from countries like Rwanda, did not show any correlation between the financial status of women and their utilisation of PNC [56]. The discrepancy may be attributed to the fact that Sierra Leone has not effectively implemented the free maternal healthcare services policy like Rwanda [17, 57] mainly due to the higher poverty levels in Sierra Leone. that make [17, 58]. At the individual level, poverty makes direct and indirect maternal healthcare access costs a huge burden while at the health system level, poverty negatively affects service delivery. This is evidenced by the low financial support of the government to health facilities with only 36.6% receiving financial support from the central government and only 4.9% ambulance ownership among health facilities [59]. Inadequate funding further affects availability of medical supplies and equipment and remuneration of healthcare providers leading to low motivation which partly explains the high staff absenteeism [58, 59]. All these factors affect access to care.

### Strengths and limitation

This study used the most recent SLDHS data with a larger sample size and higher quality, which substantially reduces the risk of sampling bias and measurement bias. We used a nationally representative sample and weighed the data for analysis and therefore our results are generalized to all women in Sierra Leone. The cross-sectional nature of the study does not confirm the definitive cause and effect relationship. The other limitation was that the SLDHS did not include information on crucial factors such as uptake on the four recommended PNC visits, male involvement, knowledge of PNC and the perceived quality of childbirth experience which could have an effect on uptake of PNC services.

## Conclusion

Factors such as type of delivery, frequency of ANC, working status, sex of household head, level of education, and having and having no problems with obtaining permission to access health care services operate at the individual and household levels. While factors such as region of residence and availability of health service providers (field health worker) operate at the health system/policy and community levels respectively. More importantly, there is need to intensify health education among pregnant women by making use of the field health workers. Reducing regional inequalities and empowering women through education will improve maternal health services.

## Supplementary Information

**Additional file 1: Supplementary file 1.** Factors associated with PNC utilisation in Sierra Leone as per the 2019 SLDHS.

**Additional file 2: Supplementary file 2.** Content of PNC received by women a per 2019 SLDHS.

## Data Availability

The data set used is openly available upon permission from MEASURE DHS website (URL: https://www.dhsprogram.com/data/available-datasets.cfm). The data set used in this study is openly available from MEASURE DHS website and the modified data set that was used for the final analysis can be availed upon request from the corresponding author.

## Abbreviations

EA: Enumeration area
AOR: Adjusted Odds Ratio
CI: Confidence Interval
COR: Crude Odds Ratio
DHS: Demographic Health Survey
SLDHS: Sierra Leone Demographic Health Survey
OR: Odds Ratio
SD: Standard Deviation
WHO: World Health Organization
ANC: Antenatal care
PNC: Postnatal care
SPSS: Statistical Package for Social Science
