# Supplementary material for: Prevalence and factors associated with utilisation of postnatal care in Sierra Leone: a 2019 national survey
Source: BMC Public Health. 2022 Jan 14;22:102. doi: 10.1186/s12889-022-12494-5 (PMC8760783; doi:10.1186/s12889-022-12494-5)
Supplement: Supplementary file 1 — Additional file 1: Supplementary file 1. Factors associated with PNC utilisation in Sierra Leone as per the 2019 SLDHS. [file 12889_2022_12494_MOESM1_ESM.docx]

**Supplementary file 1: Factors associated with PNC utilisation in Sierra Leone as per the 2019 SLDHS**

| Characteristics | Crude model  cOR (95% CI) | P-value | Adjusted model  aOR (95% CI) | P-value |
| --- | --- | --- | --- | --- |
| **Caesarean section** |  |  |  |  |
| No | 1 |  | 1 |  |
| Yes | 7.10 (3.20-15.76)* | <0.001 | 5.34 (2.23-12.82)* | <0.001 |
| **Skilled birth attendance** |  |  |  |  |
| Yes | 1 |  |  |  |
| No | 5.86 (4.60-7.47)* | <0.001 | - |  |
| **Visited by fieldworker** |  |  |  |  |
| No | 1 |  | 1 |  |
| Yes | 1.77 (1.44-2.18)* | <0.001 | 1.74 (1.40-2.17)* | <0.001 |
| **ANC frequency** |  |  |  |  |
| Less than 8 contacts | 1 |  | 1 |  |
| 8 contacts and above | 1.42 (1.12-1.81)* | 0.004 | 1.39 (1.10-1.78)* | 0.006 |
| **ANC initiation timing** |  |  |  |  |
| First trimester | 1 |  | 1 |  |
| After first trimester | 1.11 (0.93-1.32) | 0.255 | 1.02 (0.83-1.31) | 0.882 |
| **Age** |  |  |  |  |
| 35 to 49 | 1 |  |  |  |
| 20 to 34 | 1.26 (1.08-1.48)* | 0.003 | 0.99 (0.81-1.22) | 0.932 |
| 15 to 19 | 1.30 (0.98-1.73) | 0.068 | 0.91 (0.60-1.38) | 0.643 |
| **Residence** |  |  |  |  |
| Rural | 1 |  | 1 |  |
| Urban | 1.41 (1.08-1.84)* | 0.011 | 0.77 (0.50-1.18) | 0.226 |
| Region |  |  |  |  |
| **Western** | 1 |  | 1 |  |
| Southern | 1.10 (0.72-1.66) | 0.668 | 1.11 (0.68-1.83) | 0.670 |
| Northwestern | 0.48 (0.33-0.72)* | <0.001 | 0.62 (0.39-0.98) | 0.741 |
| Northern | 0.48 (0.31-0.74)* | 0.001 | 0.40 (0.23-0.68) | 0.290 |
| Eastern | 0.72 (0.48-1.08) | 0.110 | 0.56 (0.35-0.91)* | 0.019 |
| **Religion** |  |  |  |  |
| Christianity and others | 1 |  | 1 |  |
| Islam | 0.82 (0.66-1.02) | 0.075 | 1.04 (0.82-1.31) | 0.744 |
| **Sex household head** |  |  |  |  |
| Male | 1 |  | 1 |  |
| Female | 0.82 (0.68-0.99)* | 0.047 | 0.72 (0.57-0.91)* | 0.005 |
| **Household Size** |  |  |  |  |
| 7 and above | 1 |  | 1 |  |
| Less than 7 | 1.12 (0.93-1.35) | 0.231 | 1.04 (0.84-1.29) | 0.726 |
| **Working status** |  |  |  |  |
| Not working | 1 |  | 1 |  |
| Working | 0.61 (0.49-0.75)* | <0.001 | 0.67 (0.52-0.87)* | 0.002 |
| **Marital status** |  |  |  |  |
| Not married | 1 |  | 1 |  |
| Married | 0.85 (0.69-1.03) | 0.095 | 0.95 (0.74-1.23) | 0.698 |
| **Education Level** |  |  |  |  |
| No Education | 1 |  | 1 |  |
| Primary Education | 1.12 (0.91-1.38) | 0.268 | 0.87 (0.69-1.10) | 0.249 |
| Secondary Education | 1.49 (1.22-1.82)* | <0.001 | 0.97 (0.74-1.27) | 0.830 |
| Tertiary | 3.98 (2.17-7.30)* | <0.001 | 2.28 (1.07-4.83)* | 0.032 |
| **Wealth Index** |  |  |  |  |
| Poorest | 1 |  | 1 |  |
| Poorer | 1.27 (1.03-1.57)* | 0.026 | 1.29 (1.01-1.65)* | 0.039 |
| Middle | 1.66 (1.26-2.18)* | <0.001 | 1.63 (1.17-2.26)* | 0.004 |
| Richer | 1.77 (1.28-2.46)* | 0.001 | 1.63 (1.03-2.56)* | 0.036 |
| Richest | 2.05 (1.39-3.02)* | <0.001 | 1.55 (0.89-2.73) | 0.124 |
| **Parity** |  |  |  |  |
| 5 and above | 1 |  | 1 |  |
| 2-4 | 1.17 (0.99-1.38) | 0.07 | 0.98 (0.79-1.23) | 0.864 |
| 1 | 1.42 (1.17-1.74)* | <0.001 | 1.08 (0.80-1.44) | 0.621 |
| **Newspapers’ exposure** |  |  |  |  |
| No | 1 |  | 1 |  |
| Yes | 1.47 (0.90-2.38) | 0.121 | 0.84 (0.46-1.51) | 0.557 |
| **Exposure to Radio** |  |  |  |  |
| No | 1 |  | 1 |  |
| Yes | 1.31 (1.06-1.62)* | 0.012 | 1.21 (0.96-1.52) | 0.115 |
| **Exposure to TV** |  |  |  |  |
| No | 1 |  | 1 |  |
| Yes | 1.18 (0.93-1.50) | 0.178 | 0.70 (0.53-0.92)* | 0.010 |
| **Internet use** |  |  |  |  |
| No | 1 |  | 1 |  |
| Yes | 1.76 (1.17-2.66)* | 0.007 | 0.99 (0.60-1.64) | 0.984 |
| **Permission to access** healthcare |  |  |  |  |
| Big problem | 1 |  | 1 |  |
| Not big problem | 1.51 (1.21-1.90)* | <0.001 | 1.61 (1.28-2.03)* | <0.001 |
| **Distance to health facility** |  |  |  |  |
| Big problem | 1 |  | 1 |  |
| Not big problem | 1.25 (0.99-1.59) | 0.065 | 0.78 (0.59-1.05) | 0.098 |
| **Delivery place** |  |  |  |  |
| Home | 1 |  | 1 |  |
| Health facility | 7.26 (5.74-9.19)* | <0.001 | 7.34 (5.83-9.25)* | <0.001 |

*: significant at <0.05
