# Supplementary material for: Prevalence and factors associated with utilisation of postnatal care in Sierra Leone: a 2019 national survey
Source: BMC Public Health. 2022 Jan 14;22:102. doi: 10.1186/s12889-022-12494-5 (PMC8760783; doi:10.1186/s12889-022-12494-5)
Supplement: Supplementary file 2 — Additional file 2: Supplementary file 2. Content of PNC received by women a per 2019 SLDHS. [file 12889_2022_12494_MOESM2_ESM.docx]

**Supplementary file 2: Content of PNC received by women a per 2019 SLDHS**

| PNC component | Frequency | % | 95% CI |
| --- | --- | --- | --- |
| Cord examined | 6120 | 83.5 | 82.1-83.8 |
| Temperature measured | 5953 | 81.3 | 80.0-81.7 |
| Counselled on newborn dangers | 5930 | 80.9 | 79.7-81.4 |
| Counselled on breastfeeding | 6060 | 82.7 | 81.6-83.3 |
| Health provider observed breastfeeding | 5408 | 73.8 | 72.6-74.6 |
| Had all the above | 4895 | 66.8 | 65.7-67.8 |
